# Comparison of a web-based package with tutor-based methods of teaching respiratory medicine: subjective and objective evaluations

**DOI:** 10.1186/1472-6920-7-41

**Published:** 2007-11-01

**Authors:** Susan F Smith, Nicola J Roberts, Martyn R Partridge

**Affiliations:** 1 Respiratory Medicine, Imperial College London, NHLI at Charing Cross Campus, Fulham Palace Road, London W6 8RP, UK

## Abstract

**Background:**

Respiratory disease is a major cause of morbidity and mortality not only in the United Kingdom, but globally. A good understanding of respiratory disease and its treatment is essential for all medical graduates. As a result of changes in clinical practice, patients with some common respiratory illnesses are less often admitted to hospital, restricting the experience available to undergraduate students. Combined with a potential shortage of clinical teachers, this means that new methods of teaching need to be developed and appraised. The aim of this study was to establish whether a web-based package on the diagnosis of respiratory disease would be as effective and as acceptable to final year medical students as tutor-led methods of teaching the same material.

**Methods:**

137 out of 315 final year undergraduate students in a single medical school volunteered to take part. Each received up to two hours of tutor-lead interactive, tutor-lead didactic or electronic, Web-based teaching on the accurate diagnosis and management of respiratory disease. Post teaching performance was assessed by multiple true/false questions and data interpretation exercises, whilst students' teaching preferences were assessed by questionnaire.

**Results:**

Despite a high knowledge baseline before the study, there was a small, but statistically significant increase in knowledge score after all forms of teaching. Similarly, data interpretation skills improved in all groups, irrespective of teaching format, Although paradoxically most students expressed a preference for interactive tutor-lead teaching, spirometry interpretation in those receiving web-based teaching improved significantly more [p = 0.041] than in those in the interactive group.

**Conclusion:**

Web-based teaching is at least as good as other teaching formats, but we need to overcome students' reluctance to engage with this teaching method.

## Background

The United Kingdom, like many other countries, has a shortage of doctors. As a result, there has been a significant recent increase in the number of students entering medical school. Over the same time there has been a reduction in appointment of academic clinicians [1-3], an increase in pressure on clinical staff to deliver demanding service targets and the introduction of legislation limiting hours in the workplace. Together these factors have combined to reduce the number of staff available to teach. An additional problem for teachers of respiratory medicine is that many respiratory patients are treated within the community, rather than as hospital in-patients, thus potentially limiting the range of direct experience open to undergraduate and junior postgraduate trainees. Another significant pressure at present is the need in the United Kingdom to develop a coherent programme of postgraduate education in response to the recent re-structuring of clinical training in the first two postgraduate years. As a result of these combined pressures, many medical schools are developing e-learning packages which will meet current or anticipated shortfalls in undergraduate teaching and which, in addition, can be made available as postgraduate learning resources.

For the respiratory clinician, a particularly important topic for both undergraduate and postgraduate trainees is the use and interpretation of spirometry (lung function tests). Correctly used, spirometry is a powerful diagnostic tool which can also be used to monitor disease progression and the effectiveness of treatment. Despite this, published evidence suggests that spirometry is under-used both in primary care and hospital settings and is also poorly understood by clinical trainees [4-8]. In addition, a recent review of the literature has highlighted the apparent rarity of teaching on pulmonary function testing in undergraduate curricula [9].

Therefore the aim of the current study was to develop an electronic module which could be used as a stand-alone session on the correct diagnosis of respiratory illness including the role of spirometry as a diagnostic tool. To our knowledge, there have been no systematic investigations of the value of electronic tools for teaching respiratory medicine. In order to validate the module we performed a controlled trial, comparing subjective and objective performance before and after exposure to the e-module, a didactic lecture or a tutor-lead interactive session on the same academic content.

## Methods

### Subject recruitment and study design

Volunteers were recruited by a single bulk email sent in mid-February 2005 to the cohort of final year medical undergraduates due to graduate from Imperial College London in July of the same year inviting them for an additional teaching session on the diagnosis of respiratory medicine. 137 students (43% of the year cohort) volunteered to take part by e-mailing a response to the original invitation. All but 5 participants responded to the call within 72 hours, the remainder responding within 7 days. As responses were received, volunteers were allocated by a process of alternation to one of three groups, until the approximate capacity of the room used for face-to-face teaching was reached, at which point, the remaining students were allocated to the computer lab for Web-based teaching. This method of allocation was acceptable since concealment of the teaching format is regarded as more important than the actual method of participant assignment [10] and participants were allocated by one of the authors (SFS) who does not teach final year students and therefore had no personal knowledge of these students' abilities or interests. Participants themselves could not bias the process of allocation, since they had no way of knowing either the sequence in which their emails were received or the way in which the allocation was being made. Students in the three groups were taught identical content on the diagnosis of lung disease, including spirometry, but in different formats:

1. A formal didactic 90 minute lecture (n = 40).

2. An interactive 90 minute lecture by the same tutor using case studies (n = 40).

3. A customised web-based interactive learning tool using the same case studies as those used in group two (n = 57). Interactive learning materials were produced in Word and uploaded onto the Imperial College intranet using a WebCT platform. Students were allowed to access the material for two hours and study it at their own pace. This format was slightly longer than the tutor-lead formats, to allow students who might wish to revisit some parts of the material, to do so. They were given neither input from an experienced teacher nor any opportunity to ask questions of a teacher during the session, but they were permitted to discuss the material with their peers and work together if they so desired.

On being allocated to a group, students were told to which room they should report for the additional teaching. Thus, students allocated to the computer lab were able to deduce that they were likely to receive computer based teaching.

**Figure 1:**
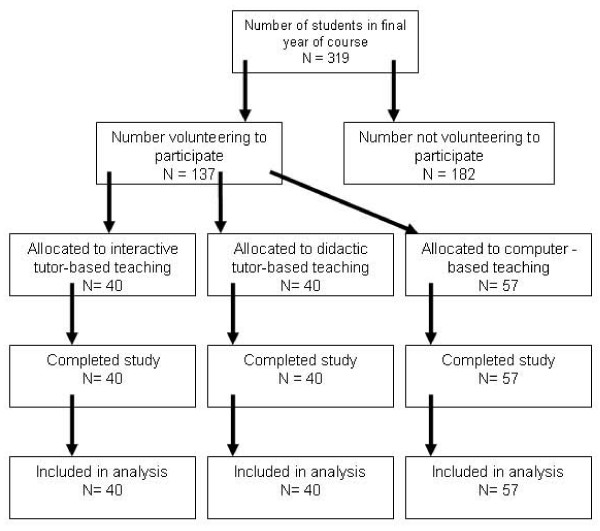
Flow chart to show numbers of students participating in trial of different formats for teaching respiratory medicine.

As both face to face sessions were led by the same person, it was not possible to deliver all teaching simultaneously. To minimise contamination (ie communication between students) the first tutor-lead session was delivered simultaneously with a computer lead session on a Friday afternoon. The second tutor-lead session and a second computer session were delivered simultaneously on the following Monday afternoon. The study design is summarised in Figure 1.

### Content of learning materials

An independent experienced respiratory teacher who was not involved in the study reviewed all learning and evaluation materials prior to use. In all cases the key learning outcomes for the teaching included the following.

On completion, the student should be able to:

• Summarise the national significance of lung disease

• Describe common respiratory symptoms

• Differentiate asthma from chronic obstructive pulmonary disease (COPD) on the basis of history and investigations including spirometry

• Know how spirometry should be performed and define key measurements of lung function

• Classify respiratory diseases into obstructive and restrictive (or small lung) disease using spirometric and other data

• Be able to interpret common spirometric abnormalities

### Assessment of student performance before and after learning session

Pre- and post-assessment of all participants was undertaken to determine the effectiveness of knowledge transfer by the three teaching formats. Participants completed the same ten multiple-true/false questions before and after their teaching session. Two control questions assessed topics which were not covered in any of the teaching formats.

The second part of the pre- and post-assessment concerned data interpretation and because of the nature of the material it was not thought appropriate to use the same data more than once. The data interpretation section which the undergraduates completed before the project therefore included five items associated with the symptom of breathlessness, followed by two sets of spirometry results for them to report. The data which the undergraduates interpreted after the teaching session included three causes of breathlessness and three examples of spirometry.

All students were required to complete the assessments independently, without conferring with colleagues.

All scripts were marked blind using a pre-prepared mark scheme. Differences in knowledge transfer were analysed using ANOVA followed by 2-tailed non-parametric Mann-Whitney tests for each individual item. Since different questions were asked before and after teaching, the effect of teaching within a group was analysed by expressing marks as percentages both for each question and for the entire test and analysing the percentages. Differences in performance on the data interpretation questions between groups were analysed using Repeated MeasuresGeneralized Linear Models.

### Student evaluation

Finally, at the end of the teaching session, all students were asked to identify their preferred learning format and were asked a series of open questions to elucidate their views about the format of teaching they had experienced. Twenty-five percent of responses were themed by the two lead investigators (SFS and MRP) and comments relating to major themes enumerated for all participants [11].

### Ethical approval

This study was approved by the Head of Undergraduate Medicine, Faculty of Medicine and the Head of the National Heart and Lung Institute Division of Imperial College London.

## Results

There was no difference in any outcome between the two computer lead groups, suggesting that there was little communication between these groups between sessions. These data were therefore pooled and analysed as a single group. Although access to the E-learning modules was for the same time period for all students, the ways in which they used the package varied enormously. Time actually spent logged onto the relevant webpages varied from less than 5 minutes to the full two hours allocated. Those on line for a very short time were probably working with a colleague, so only one of them remained logged in. Forty six students were logged on for less than 60 minutes in total, 9 for 60 – 120 minutes and 2 for more than 120 minutes. Similarly, whilst some students remained logged in throughout their use of the material, others logged in and out, visiting other sites during the session. The maximum number of hits by one student was 11 in a period of 59 minutes.

### Assessment of student performance

**Figure 2:**
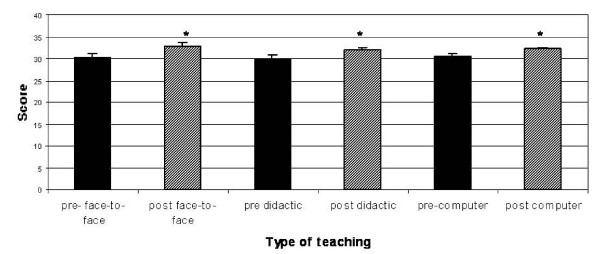
**Effect of teaching format on overall knowledge transfer**. One mark was given for each correctly answered item; maximum score was 52. All groups showed a small, but statistically significant increase in score for taught items only. Data are expressed as means ± standard deviation. * > same group before teaching, P < 0.05

Prior to the teaching, there were no significant differences between groups in the scores for the multiple true/false questions (Figure 2). After teaching, the overall scores for all three groups showed a modest, but significant improvement amounting to an average of two or three additional correct responses (Figure 2). Scrutiny of results for individual questions shows that there was no change in scores for the two questions concerning subjects not taught during the educational sessions (Figure 2).

**Figure 3:**
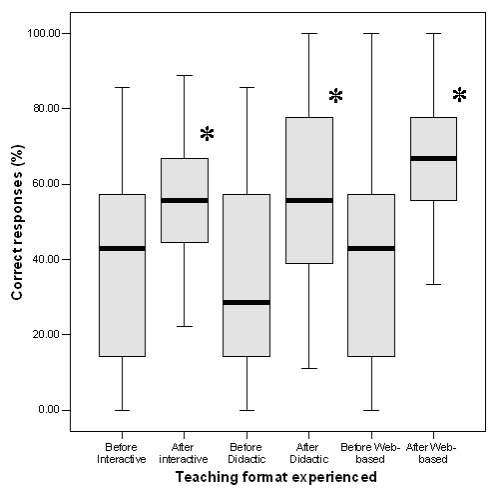
**Effect of teaching on total data interpretation scores for each teaching format**. Data were expressed as percentages of the maximum possible score before and after teaching and are illustrated as medians bisecting the 25 and 75% percentiles and the limits of the range. There were no differences in performance between groups prior to teaching. All groups showed a significant improvement. Analysis by general linear model with repeated measures showed a significant improvement in performance after teaching (*: P < 0.001 in all cases).

**Figure 4:**
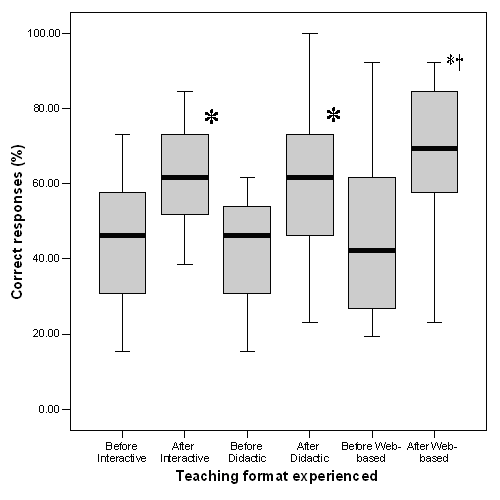
**Effect of teaching on scores for interpretation of spirometry reports for each teaching format**. Data were expressed as percentages of the maximum possible score for spirometry questions before and after teaching and are illustrated as medians bisecting the 25 and 75% percentiles and the limits of the range. There were no significant differences in performance between groups prior to teaching. All groups showed a significant improvement in performance after teaching (*: P < 0.01 in all cases). After teaching, students who had experienced the Web-based package performed significantly better than those who had experienced the tutor-lead, interactive teaching (†: P = 0.041).

The summary of results for the overall scores in data interpretation, pre and post teaching, are shown in Figures 3 and 4. This shows that prior to teaching, there were no differences between the groups in understanding of the causes of breathlessness (Figure 3) or in the capacity to interpret spirometry (Figure 4). There was a significant improvement in all groups after teaching (p < 0.001), the improvement being slightly, but not significantly greater in the computer-based group compared to improvement by the students receiving interactive (p = 0.079) or didactic (p = 0.058) tutor-lead teaching. Further analysis of the individual scores show that those having web-based education demonstrated a markedly improved ability to interpret spirometry (Figure 4) which was significantly greater than that of students in the interactive group (p = 0.041) and slightly, but not significantly greater than students in the didactic group (p = 0.064). This was particularly noticeable in one specific example which included a flow volume curve showing the classical appearance of extra-thoracic airway obstruction.

### Student evaluation of teaching formats

**Figure 5:**
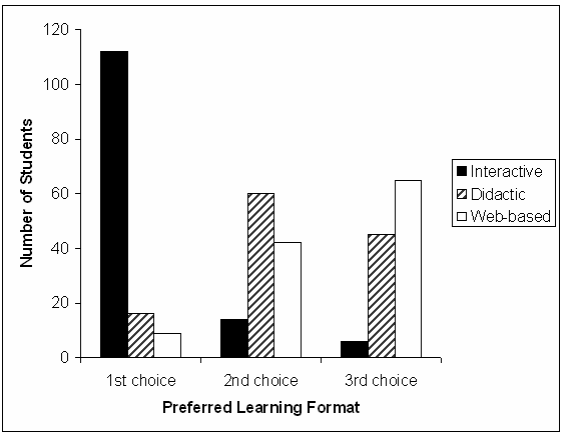
**Preferred learning formats of final year medical undergraduates**. Histogram showing the first, second and third choice preferences for the entire cohort of participants. The overwhelming majority identified tutor-lead, interactive teaching as their preferred format, irrespective of the teaching format they had just experienced.

**Figure 6:**
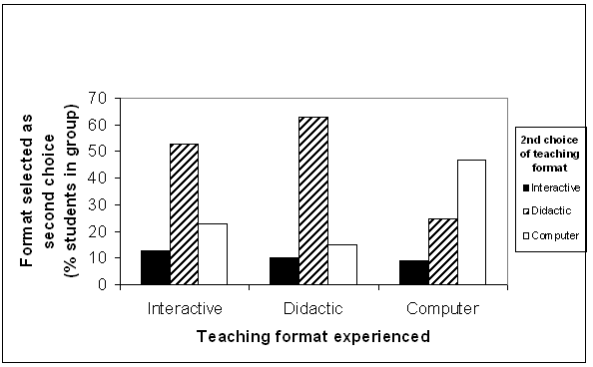
**Impact of teaching format experienced in study on expression of second choice of teaching format**. Students who had experienced the web-CT based teaching package were more likely to put web-based teaching as their second choice compared with those who had received tutor-lead teaching.

All students found their sessions useful, irrespective of format. When asked to identify the format they would have chosen, had they been given a free choice in this study, the majority identified interactive tutor-lead learning as their preferred format (Figure 5). When asked why they would have selected this format, the commonest reasons cited were that the possibility of being asked a question acted as an aid to concentration (53 comments) and the presence of the tutor provided an opportunity to ask questions about areas of difficulty (67 comments) although, interestingly, relatively few elected to do so on this occasion. This was not a universal view though. Students expressing a preference for a didactic lecture (n = 16) liked the faster pace when the flow of information was not interrupted by student questions (5 comments) and found the lack of tutor-interaction less threatening than the possibility of being asked a question (4 comments). For example "it can prevent me from taking in what's been said as I become nervous of being asked (often despite knowing the answer!)". In addition, 9 students would have preferred the computer based format, eight of them citing the ability to self-pace as their main reason (e.g. "I can take my time and return to areas not properly understood"). Students who had experienced the web-CT based package were more likely to put computer-based teaching as their second choice, compared to those students who had received tutor-lead teaching during the study (Figure 6).

When asked specifically what they liked and disliked about the teaching format they had experienced in the study, many of the students who had been allocated to the computer group reported liking it because it was self-paced (38/57) and because they were able to re-visit material at will (20/57). They disliked the lack of opportunity to ask questions or to pursue an area of interest in more depth (23/57) and several (10/57) commented that they disliked spending a long time looking at the computer screen. In contrast, negative comments about the tutor-lead teaching formats tended to focus on the physical environment (room too warm, uncomfortable chair) with very few comments about the teaching itself.

When asked after the session to list the specific items they could recall from the teaching, most participants from all groups noted points relating to spirometry (123/137), and the classification of lung disorders and the causes of breathlessness (104/137). A significant number also recalled information given on the epidemiology of lung disease (60/137)

## Discussion

In this study, we demonstrated a small, but statistically significant increase in level of knowledge, and a substantial significant improvement in data interpretation skills after all three teaching methods. An improvement in spirometry interpretation was particularly marked in the group experiencing web-based teaching and underlay the overall improvement in data interpretation skills in all three groups. We sought to minimise the risk of students confounding the results by private study by assessing learning immediately after the session, and by assessing enhanced understanding in addition to knowledge transfer.

Using this study design, we demonstrated that despite interactive, face-to-face teaching being the preferred option of the majority of participants, short-term knowledge transfer and a marked improvement in data interpretation skills was equally achievable by all three methods of teaching. One noteworthy feature of the study was that the participants were students only four months away from their final examinations, who had high baseline knowledge prior to the teaching and thus, the margin for improvement in knowledge was small. Despite this, a significant improvement was detected in performance on taught items in all groups. Furthermore, there was no improvement in performance on untaught items, indicating that all three learning formats were of value in this situation. This finding suggests that computer-based teaching may be particularly valuable for senior students who need to consolidate and extend their knowledge in respiratory medicine; whether it is equally suitable for novices in this field needs to be established. We elected to study students at this time, because previous experience has shown them to be eager for additional teaching and thus, it was possible to recruit a substantial number of participants (40% of year group).

To our knowledge this is the first trial designed specifically to examine the effectiveness of a computer-based package at facilitating an improvement in respiratory data handling skills. In line with current findings, most studies designed to investigate educational questions have shown only modest differences or no difference between teaching formats in knowledge transfer [12-24] whilst those considering attitude tend to show a preference for tutor-directed teaching methods even when computer packages are highly rated. However, the ability to interpret lung function data is a crucial skill in respiratory medicine so we were particularly anxious to determine the efficacy of computer-based teaching for this purpose. The study shows that, in this respect, it is at least equivalent to other teaching formats and in this cohort, superior to face-to-face interactive teaching. Close analysis of the data demonstrated that the improvement in performance of the students receiving web-based teaching was largely due to their ability to interpret one particular lung function report illustrating extra-thoracic airway obstruction. This was portrayed along with other examples during both face to face teaching and in the web-based package. However, because this condition is uncommon, the lecturer devoted less time to it than to other, more common, conditions. Our observation suggests that students studying independently weight all the information given equivalently and thus, were more likely to recall a less common area. If this interpretation is correct, it demonstrates the importance of ensuring that materials prepared for private study by students should be focussed on core areas to avoid them being overwhelmed by interesting, but non-core material. We know that the students remained logged on to the e-learning materials for variable lengths of time and whilst some remaining logged on throughout the session, others logged in and out up to 11 times. We do not know, of course, whether they were actually working whilst logged in, or that they had stopped working because they had logged out – they may have been accessing other relevant materials, but the log does demonstrate that the students did personalise their use of the materials, which is one of the strengths of this teaching format. Of further interest is that benefit was gained from this method of teaching despite few students needing the full time period which we had allocated. Indeed many spent less time on the project than those taking part in the tutor lead sessions.

Student preference or perception of performance does not always correlate with objective measurements. In a British based study concerning the teaching of psychiatry Williams *et al *[21] found that although students rated their learning as greater in a lecture on panic and anxiety than from a computer package on the same subject, objective assessment showed equivalence of knowledge transfer. Student choice of learning format is likely to be determined by a number of factors including individual learning styles and previous experience. It is likely that many of the participants in our study had only limited experience of interactive, web-based educational methods, since these techniques were not widely used in our undergraduate course at the time of this study. Sub-group analysis of the preferences, showed that in the group that received the web-based teaching, participants were more likely to make electronic teaching their second choice (after interactive tutor-lead) compared with the other two groups. (Figure 6). This bodes well for the future suggesting that, as they become familiar with these, students find e-learning packages more acceptable. Indeed, one respondent who selected e-learning as his preferred format stated this explicitly – "I might not have chosen computers initially, but it was very good."

Advantages of web-based teaching are that it is self-paced and also permits the user to review topics at will – points that were made by a number of our students in their evaluations. However, students studying independently are less likely to receive the benefit of feedback [25]. A partial solution to this problem may be to make modules available for a set time, inviting email questions arising from the module and posting anonymised questions and answers on a virtual notice board. This would permit the tutor to schedule time to answer questions whilst allowing reasonable immediacy of feedback to students. Frequently asked questions could then be used to make improvements to the module for future students. The use of a more interactive form of computer based learning has been shown to improve knowledge retention on neuroanatomy and neurophysiology compared to a computer package with more didactic content.

Whilst it can never replace the inspiration engendered by interaction with an enthusiastic expert teacher, web-based teaching offers the ability for all learners to study flexibly and with repetition as often as they wish. An ideal outcome would be the combining of both formats, so web-based teaching is embedded within the curriculum, for example, by using a face-to-face session to de-brief students following a web-based activity. Previous (unpublished) experience at our institution has shown that offering e-learning opportunities without firmly embedding them in some way, results in very poor use of web-based resources. This study adds further information to reassure us that, at least for teaching senior students, such methods can be as effective as existing techniques and can be used to teach data interpretation skills, as well as knowledge transfer. Whether this is equally true for novice learners remains to be established.

## Conclusion

### What is known about this area

Most randomised controlled trials of different learning methods show no difference in knowledge transfer between study groups, but, despite this, students often express a preference for tutor-directed learning formats.

### What this study adds

A computer-based package is at least as effective as tutor-directed methods for enhancing students' data interpretation skills relating to the diagnosis of respiratory disease.

### Suggestions for future research

Future studies should investigate the students' approach to self-directed, computer based learning and establish how they decide to apportion their time and attention between the plethora of material available to them. This would help teachers prepare better materials for student use.

## Competing interests

The author(s) declare that they have no competing interests.

## Authors' contributions

MRP and SFS had the original idea for the development of the material, MRP undertook the teaching and SFS organised the study. All authors interpreted the data and wrote this report.

## Pre-publication history

The pre-publication history for this paper can be accessed here:


